# The Safety of Bilateral Simultaneous Hip and Knee Arthroplasty versus Staged Arthroplasty in a High-Volume Center Comparing Blood Loss, Peri- and Postoperative Complications, and Early Functional Outcome

**DOI:** 10.3390/jcm10194507

**Published:** 2021-09-29

**Authors:** Michael Najfeld, Thomas Kalteis, Christian Spiegler, Christophe Ley, Robert Hube

**Affiliations:** 1 OCM Orthopädische Chirurgie München, Steinerstr. 6, 81369 München, Germany; thomas.kalteis@ocm-muenchen.de (T.K.); christian.spiegler@ocm-muenchen.de (C.S.); robert.hube@ocm-muenchen.de (R.H.); 2 Department of Applied Mathematics, Computer Science and Statistics, Ghent University, 9000 Ghent, Belgium; christophe.ley@ugent.be

**Keywords:** results, complications, specialized center, joint replacement

## Abstract

Purpose: In recent years, there has been increasing interest in the use of simultaneous hip and knee arthroplasty compared to staged procedures in patients with bilateral pathology. The aim of this study was to compare simultaneous and staged hip and knee arthroplasty in patients with bilateral pathology by assessing the transfusion rate, postoperative hemoglobin drop, length of stay (LOS), in-hospital complications, 30-day readmissions and early functional outcome. Methods: We conducted a retrospective cohort study that included all patients who were undergoing primary TKA, THA and UKA by a single surgeon in a high-volume arthroplasty center between 2015 and 2020 as simultaneous or staged procedures. Staged bilateral arthroplasties were performed within 12 months and were stratified by the time between procedures. Data were acquired through the electronic files at the Orthopädische Chirurgie München (OCM). For functional outcome, the ability of the patients to walk independently on the ward was compared with the ability to walk a set of stairs alone, which was recorded daily by the attending physiotherapist. Results: In total n = 305 patients were assessed for eligibility and included in this study. One hundred and forty-five patients were allocated to the staged arthroplasty group. This group was subdivided into a hip and a knee group, whereas the knee group was split into TKA and UKA. The second staged procedure was performed within 12 months of the first procedure. One hundred and sixty patients were allocated to the simultaneous arthroplasty group. This group was also subdivided into a hip and knee group, whereas the knee group was split again into a TKA and UKA group. No statistical difference was found between the two groups regarding demographic data. Primary outcome measurements: There was no significant difference in the transfusion rate or complication rate. Secondarily, no statistically significant difference was found between the postoperative hemoglobin drop and the functional outcome, or in the length of stay (LOS) between both groups. Walking the stairs showed a significant difference in the knee group. Conclusions: There were no significant differences observed in the transfusion rate in-hospital complications, or readmission rate between both groups. The early functional outcome showed no significant difference in mobility for all groups. Simultaneous arthroplasty for knee or hip is as safe as a staged procedure, with no higher risk for the patient, in a specialized high-volume center. Level of evidence: Level IV.

## 1. Introduction

Bilateral simultaneous total hip arthroplasty (THA), as well as bilateral simultaneous total knee arthroplasty (TKA), has been on the rise in recent years. Initial data suggested, however, that simultaneous bilateral hip arthroplasty is associated with an increased rate of complications, including occurrences of deep-vein thrombosis (DVT) [1], as well as increased medical morbidities, thus leading to suboptimal functional outcomes [2]. Other studies found increased postoperative mortality and complications after simultaneous bilateral TKA compared to staged bilateral TKA, with pulmonary embolism and cardiac complications as the major complications [3,4,5,6]. In contrast, other studies reported no differences in mortality and complications between simultaneous and staged procedures [7,8,9]. The aim of this study was to show the safety and effectiveness of a bilateral simultaneous procedure.

This led to the evaluation of the best strategy to operate on a patient presenting with bilateral hip or knee pathology without increasing the risk of perioperative comorbidities, with options such as simultaneous bilateral hip or knee replacement or a sequential surgery [10]. From the perspective of fast-track surgery, it would be ideal to offer a simultaneous bilateral procedure instead of a staged procedure. Promising results with low postoperative morbidity and mortality after simultaneous bilateral TKA have been reported in selected patients [11,12,13].

However, systemic reviews of THA and TKA were not always conclusive for simultaneous versus staged arthroplasty [14,15]. This study aimed to compare the safety of bilateral simultaneous THA and TKA versus staged arthroplasty in a high-volume center. We hypothesized that there was no difference in outcome between these two options.

## 2. Patients and Methods

The regional ethics committee waived the need for approval on 20 January 2021, as this was an observational non-interventional study. We conducted a non-randomized, non-blinded retrospective cohort study that included all patients scheduled to undergo primary TKA, THA and UKA by one single surgeon (R.H.) in a high-volume arthroplasty center between January 2017 and December 2020. The inclusion criteria were any patient undergoing a primary TKA, THA and UKA for primary or secondary knee osteoarthritis without previous joint arthrotomy. Exclusion criteria were patients that lacked capacity to consent to the study, patients who were unwilling to consent and patients with previous joint arthrotomy, as well as revision cases.

All surgeries were performed without a tourniquet with the medial parapatellar approach for TKA, OCM-approach (muscle sparing anterolateral approach) for THA and a modified minimally invasive parapatellar approach for the UKA. All departments have implemented protocols, including for anaesthesia, tranexamic acid (1 g intravenous preoperatively and 3 g intraarticular), opioid-sparing analgesia with acetaminophen, non-steroid anti-inflammatory drugs (NSAIDs), and early mobilization, regardless of age or preexisting comorbidity. The postoperative rehabilitation regimens were the same for both groups and started on the day of surgery. The first mobilization of the patient for all joints was performed under the supervision of physiotherapy in the postoperative area or on the regular ward. Transfer from the bed to the floor and quadricep-setting exercises were started on day 1 after surgery. Ambulatory training with weight bearing as tolerated and active-assisted range of motion exercises was started on the day of surgery as well as gait training using a walker. Walking outside on the ward was facilitated as soon as the patient was ambulating safely in his room and stair assessment was performed as soon as free walking outside was performed.

Demographic data such as age, sex, height, weight and the resulting body mass index and operated side were collated. Operation time (OR time), surgical and medical complications were collated from the patient’s electronic file, as well as every visit of the patient within 90 days postoperatively to the outpatient clinic. The OR time was defined by the incision of the skin and the closure of the skin by the last suture.

**Figure 1:**
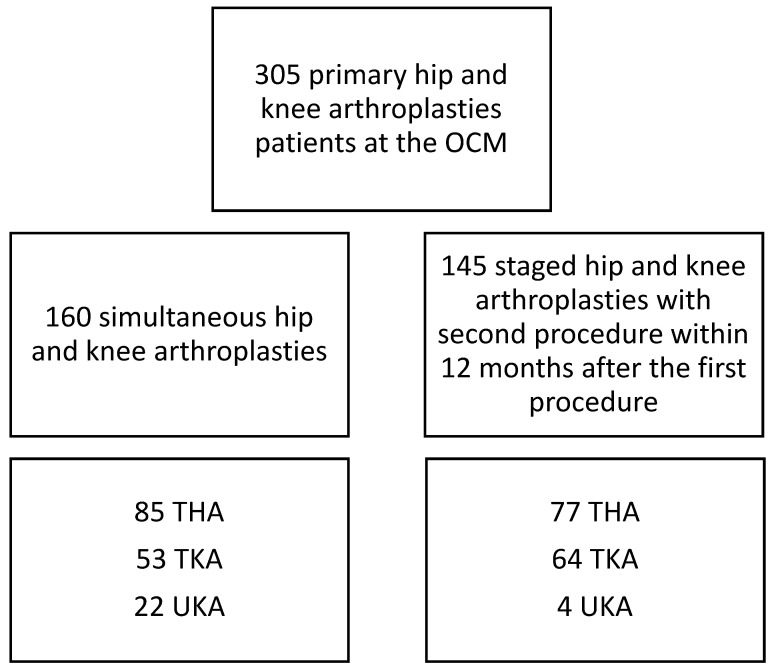
Flowchart of the study’s selected patients. THA: Total hip arthroplasty, TKA: Total knee arthroplasty, UKA: unicompartmental knee arthroplasty.

In total *n* = 305 patients were included in the study. The included patients were divided into 2 groups according to how the arthroplasty was performed, simultaneous *n* = 160 (52.46%) versus staged *n* = 145 (47.54%). Within each group, patients were differentiated between THA, TKA and UKA. Figure 1 shows the separation into the specific groups.

Correlations have been calculated via the classical Pearson correlation coefficient and the associated uncertainty is conveyed via confidence intervals.

The postoperative Hemoglobin drop between the two groups was compared via the Kolmogorov–Smirnov two-sample test, while all other comparisons have been achieved via the Mann–Whitney U test (also known as Wilcoxon rank-sum test). We made this distinction because Hemoglobin values were given at a continuous scale, contrary to the discrete ordinal scale of all other values (LOS, Stairs POD, free-moving POD, OR time). The significance level of all tests was 5%.

## 3. Results

The study population consisted of *n* = 305 patients. One hundred and sixty patients received a simultaneous procedure versus *n* = 145 with staged arthroplasty. In the THA group, *n* = 85 (53%) were simultaneous and *n* = 77 (53%) staged arthroplasties were performed. There was no significant difference in the demographic data (Table 1, Table 2 and Table 3).

**Figure 2:**
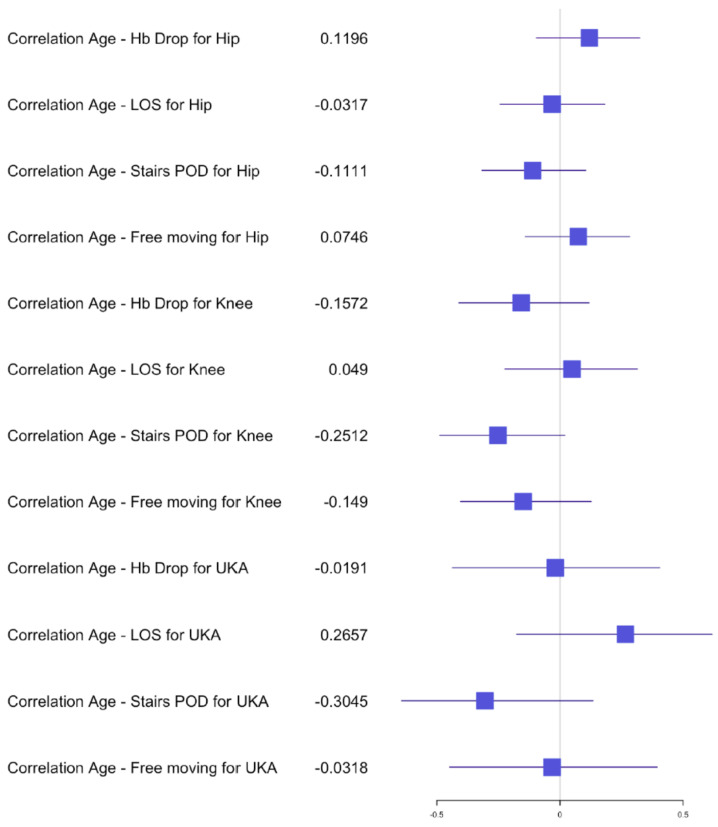
Forest plot for the effect of age on Hb drop, LOS (Length of stay) and functional outcome for simultaneous arthroplasty.

**Figure 3:**
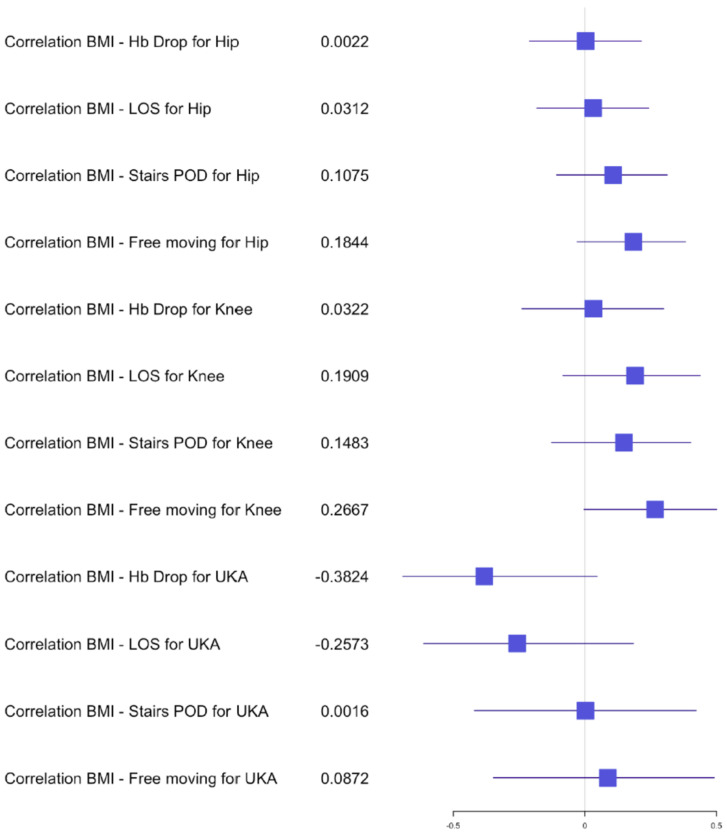
Forest plot for the effects of BMI on Hb drop, LOS and functional outcome for simultaneous arthroplasty.

**Figure 4:**
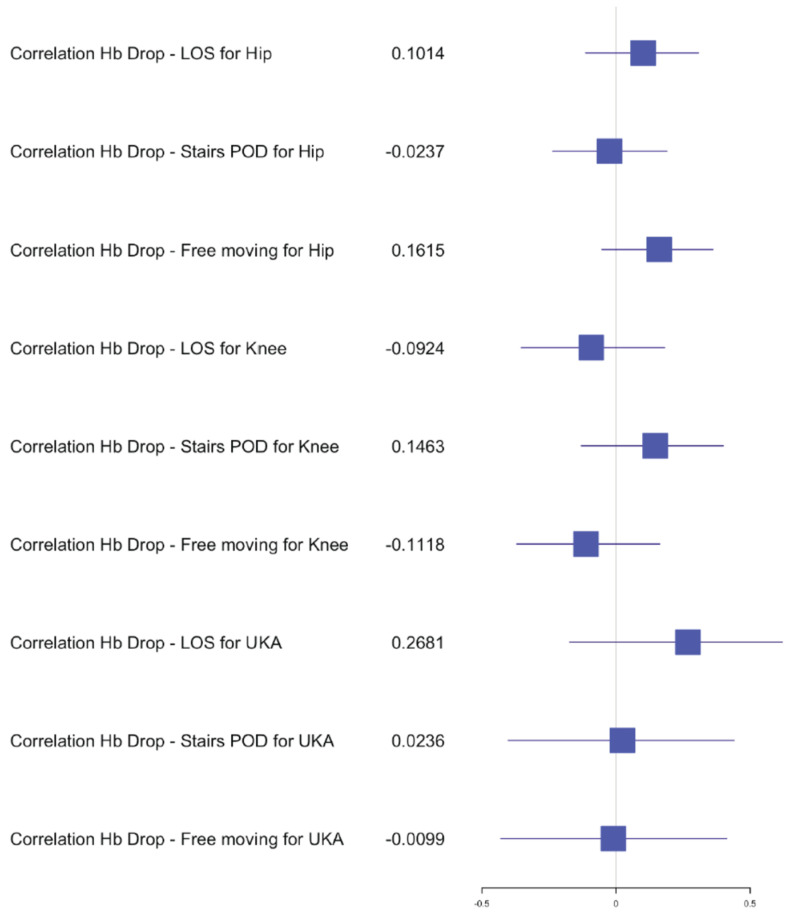
Forest plot for the effects of Hb drop on LOS and functional outcome for simultaneous arthroplasty.

**Figure 5:**
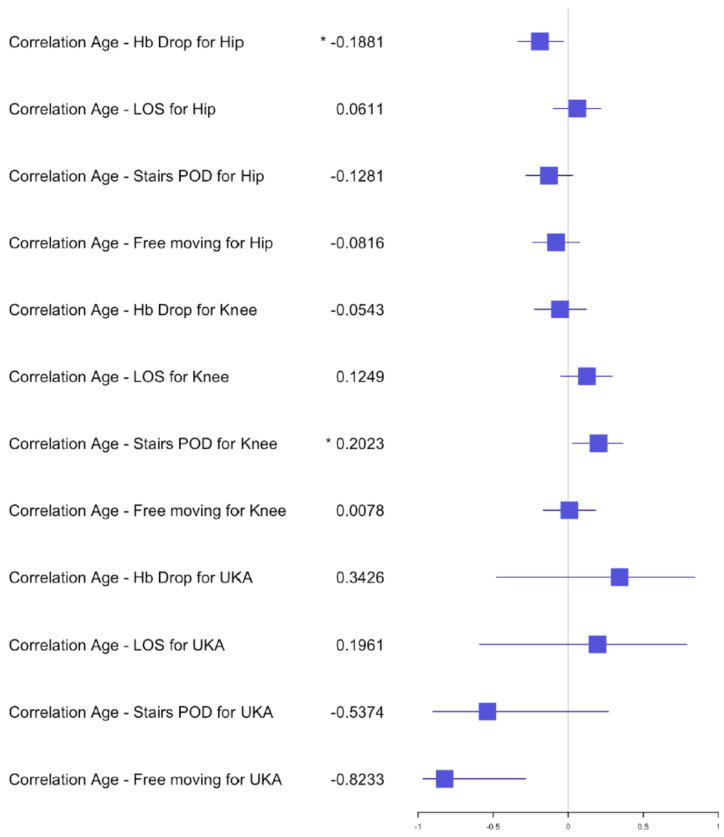
Forest plot for the effects of age on Hb drop, LOS and functional outcome for staged arthroplasty. (*) significant results.

**Figure 6:**
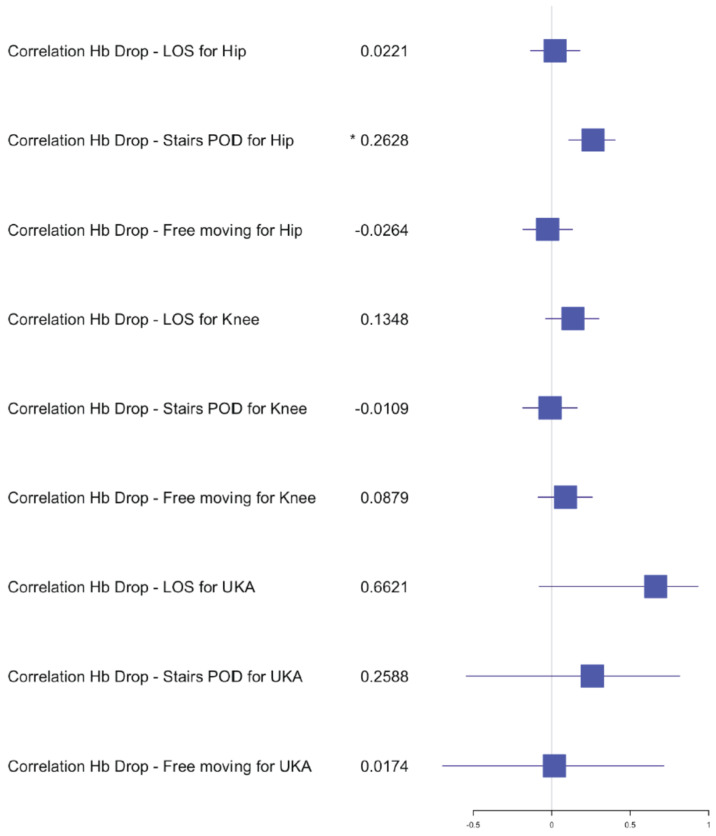
Forest plot of the effects of Hb drop on LOS and functional outcome for staged arthroplasty. (*) significant results.

The average length of stay (LOS) for the simultaneous THA group was 7 days (±2) with a range of 4–12 days, versus the staged THA group 7 days (±1.5) with a range of 3–15 days. The postoperative hemoglobin drop in the simultaneous group was, on average, 2.2 g/dL (±0.9) compared to 1.9 g/dL (±0.9) in the staged group. The transfusion rate for both groups was 0% and no significant difference was found in the postoperative hemoglobin drop after the first staged procedure (*p* = 0.1147) compared to a significant difference after the second procedure (*p* = 0.0271) in comparison with the simultaneous procedure. The average of the preoperative hemoglobin for staged procedure was 13.9 g/dL (±1.2) with a range of 10.4–17 g/dL and of the average postoperative hemoglobin was 12 g/dL (±1.2) with a range of 9.3–15.3 g/dL. The average preoperative hemoglobin for simultaneous procedure was 14.2 g/dL (±1.2) with a range of 12–16.9 g/dL and the average postoperative hemoglobin was 12 g/dL (±1.3) with a range of 9.1–14.3 g/dL. The mean OR time for the simultaneous group was 79 min (±13) versus 29 min (±5) for one hip of the staged group. Statistical analysis of OR time showed a significant difference for the hip group *p* < 0.05 dividing the OR time in half for the simultaneous procedures compared to the average of the staged procedures. The 30-day readmission rate was 0.02% after simultaneous versus 0.01% after staged THA arthroplasty. One patient had to be re-operated for a hematoma in the simultaneous group and one had to be readmitted for swelling and hematoma compared to one readmission for fracture in the staged group. No medical complications and no deaths occurred within 30 days of surgery for the THA group. Independent mobilization of the patient on the ward was achieved after 2.5 (±0.7) days and the ability to walk stairs alone was found to be 4 (±1) days for the simultaneous THA, compared to 2.5 (±0.7) days independent mobilization and 3.5 (±1) days for walking stairs alone in the staged THA group. No significant difference was found in the early functional outcome for mobility and the ability to walk stairs for the hip group. In the correlation examination, no significant difference could be found for postoperative hemoglobin drop, age and BMI in relation to LOS, functional outcome for the simultaneous group (Figure 2, Figure 3 and Figure 4). There was a significant negative correlation between age and postoperative hemoglobin drop (Figure 5) whilst in the staged group, a positive correlation with significance was described between postoperative hemoglobin-drop and the ability to walk stairs (Figure 6).

In the TKA group, *n* = 53 (33%) simultaneous versus *n* = 64 (44%) staged arthroplasties were performed. The average LOS after simultaneous TKA was 8 days (±2 days) versus 7 (±2 days) after staged procedure, with a range of 4–16 days and 3–13 days, respectively. The mean postoperative hemoglobin drop after the simultaneous procedure was 2.4 g/dL (±0.8) versus 1.9 g/dL (±0.7) after staged procedures. No transfusion was performed in both groups. The postoperative hemoglobin drop difference after the first and second staged procedures was significant compared to the simultaneous procedure (*p* = 0.0013 and *p* = 0.0220). The average hemoglobin preoperative for staged procedure was 14.1 g/dL (±1.2) with a range of 11.3–16.8 g/dL and the average postoperative hemoglobin was 12.2 g/dL (±1.2) with a range of 10–15 g/dL. The average hemoglobin preoperative for simultaneous procedure was 14.1 g/dL (±1) with a range of 12.2–16.1 g/dL and the average postoperative hemoglobin was 11.7 g/dL (±1.2) with a range of 8.7–13.8 g/dL. The mean OR time after simultaneous TKA was 85min (±17) compared to 40 min (±10) for one TKA in the staged group. Statistical analysis showed no difference between the time taken for a simultaneous procedure and the time taken for a staged procedure. The 30-day readmission rate was 0.02% after simultaneous TKA versus 0.01% after staged TKA. One patient in the simultaneous group had to be readmitted for wound issues with no surgical treatment and 1 patient in the staged group was readmitted for early infection with surgical debridement. Within the first 2 months post-surgery, 2 patients from the simultaneous TKA group had to undergo a mobilization of the knee and an arthroscopic debridement. No medical complications and no deaths occurred within 30 days of surgery for the TKA groups. The independent mobilization of the simultaneous TKA patients was achieved after 3 days (±1.5) and the ability to walk stairs alone after 4 days (±1) versus independent mobilization after 2.5 days (±0.7) and stairs after 3.5 days (±1) in the staged TKA group. There was no difference for the independent mobilization but a significant one for the ability to walk stairs when comparing the first and second staged procedures to the simultaneous group (*p* = 0.0020 and *p* = 0.00001). In the correlation examination, no significant difference could be found for postoperative hemoglobin drop, age and BMI in relation to LOS, functional outcome in the simultaneous group (Figure 2, Figure 3 and Figure 4). In the staged group, a positive significant correlation between age and the ability to walk stairs was found (Figure 5) and a negative significant correlation between BMI and the ability to walk independently (Figure 6).

**Figure 7:**
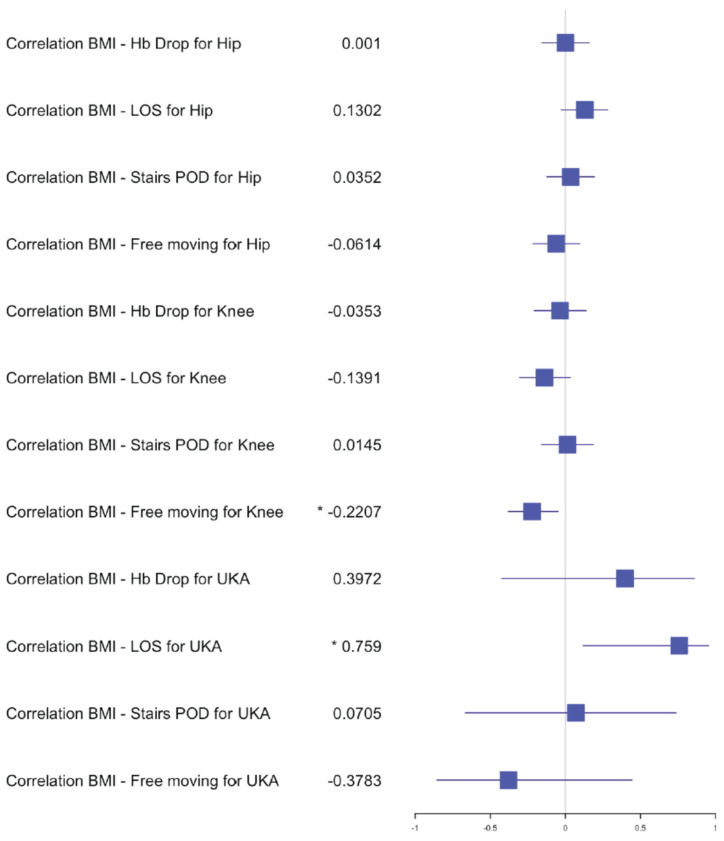
Forest plot for the effects of BMI on Hb drop, LOS and functional outcome for staged arthroplasty. (*) significant results.

In the UKA group *n* = 22 (14%) procedures were performed simultaneously compared to *n* = 4 (3%) staged. The average LOS after simultaneous UKA was 6.5 days (±2 days) versus 5.5 (±1.5 days) after staged procedure with a range of 4–11 days and 4–8 days, respectively. The mean hemoglobin drop after simultaneous procedure was 2 g/dL (±1) versus 1.5 g/dL (±0.6) after staged procedure. No transfusion was performed in both groups. The average preoperative hemoglobin for staged procedure was 14 g/dL (±1.5) with a range of 10.9–15.8 g/dL, and the average postoperative hemoglobin was 12 g/dL (±1.2) with a range of 10–13.8 g/dL. The average hemoglobin for simultaneous procedure was 15 g/dL (±1) with a range of 13.6–17.2 g/dL, and the average postoperative hemoglobin was 13 g/dL (±1.2) with a range of 10.7–15.1 g/dL. The mean OR time after simultaneous UKA was 75 min (±21) compared to 35 min (±3) for one UKA in the staged group. No patient had to be readmitted 30 days post-surgery. One patient in the simultaneous UKA group had to be postoperatively treated by internal medicine for hemorrhagic gastritis. Early independent mobilization was achieved after 2.5 days (±1) and independent mobility on stairs was achieved after 4 days (±1) in the simultaneous UKA group versus after 2 days (±1) for independent mobility for stairs and 3.5 days (±1) for stairs in the staged UKA group. No statistical analysis comparing the difference of Hb-drop, LOS and functional outcome could be performed due to the limited number of patients. In the correlation analysis, no significance could be found between age, BMI, Hb-drop compared to LOS and functional outcome for the simultaneous group. In the staged group only, a significant positive correlation was found between BMI and LOS (Figure 7).

## 4. Discussion

Our study found no significant differences between transfusion rate, peri- and postoperative complications, early functional outcome except stairs for knees, and 30-day readmission rate in THA and TKA as well as UKA between the simultaneous and stage groups. Our study found no significant differences between transfusion rate, peri- and post-operative complications, early functional outcome except stairs for knees, and 30-day readmission rate in THA and TKA as well as UKA between the simultaneous and stage groups, thus confirming the hypothesis.

The postoperative hemoglobin drop was significantly different for knee arthroplasty when comparing staged and simultaneous procedures. Nevertheless, the clinical outcome in terms of patient mobility showed no difference. The ability to walk independently on stairs was significantly delayed for knees and the positive correlation between age and stairs shows that older people have more difficulties. These finding reflect the previous published literature [16,17,18,19]. No blood transfusion was required for any patients in this study. The early mobilization on the same day of surgery likely contributed to these findings [11]. Nevertheless, for THA patients, a higher hemoglobin-drop delays the ability to walk stairs in the correlation analysis. Age, on the other hand, negatively influences the hemoglobin-drop for THA patients. No other in-hospital complications were noted to correlate with outcomes for THA, TKA and UKA.

Within the first 12 months, only (*n* = 2) patients needed additional surgery for mobilization and arthroscopic debridement in the simultaneous group compared to the staged TKA. No higher morbidity or mortality rate was found in either group. No medical complication, consisting of a pulmonary embolism, deep venous thrombosis or respiratory distress, could be found. These findings are confirming the previous meta-analysis by Haverkamp et al. [20] Only (*n* = 1) patient developed a hemorrhagic gastritis in the early postoperative phase in the simultaneous UKA group. 

The LOS in this study cannot be compared to previous studies, as the German health system has a minimum stay criterion of 3–4 days for full remuneration. Nevertheless, within this study, no significant difference could be shown within all the groups and the length of stay and no correlation was found with the BMI, age or Hb-drop. Therefore, the cumulated postoperative rehabilitation period after the simultaneous procedure can be considered shorter than the staged procedure. Early independent mobilization could be achieved at a similar time. An earlier return to normal physical activity and better quality of life can be assumed. Further investigations should be made into the quality of life and mobilization with 12 months post-surgery [16].

A higher infection rate was not observed for the THA, TKA or UKA group. Interestingly, as the OR time for knee arthroplasty per single staged procedure showed no significant difference compared to the simultaneous group and the average OR time does not exceed the 100 min windows for increased infection rate [21], it is safe to be performed. For hip arthroplasty, the significant difference can be explained by the fact that the hip is in a lateral position and, for the second procedure in the simultaneous group, the patient had to be repositioned and re-draped for the second procedure. The OR time was still running at that time. Nevertheless, in these cases, the average OR time still did not exceed the 100 min window. Different perioperative implantation, as well as the fact that these surgeries are performed by a high-volume surgeon, contribute to the reduced OR time and the mobilization on the same day of surgery.

The limitation of this study is the retrospective data collection and the number of patients in each group. Specific comorbidities are not registered and should ideally have been incorporated. This study is the first of its kind, but suffers from a limitation. Some form of selection bias has occurred: the simultaneous operation was mainly given to patients with a better health status. Since this data are retrospective and concrete comorbidities are missing, it was not possible to undertake propensity score matching. This is foreseen as a subject for a more detailed follow-up study. The results obtained in the present study are very encouraging in this direction. The UKA group was limited in its statistical analysis due to the reduced number of patients in one group. A strength of the study was the standardized fast-track setup for one high-volume surgeon for all procedures, with data collected recently. Similar to Sheth et al. [17], the second procedure of the staged group could have been performed up to 12 months after the first procedure. This would have allowed the study to include a higher number of patients. The initial plan of logistic regression for risk factors is not recommend, as the numbers are not high enough and distorted viewpoints could be reached.

## 5. Conclusions

When comparing simultaneous to staged hip or knee arthroplasty, no higher rate of transfusion, postoperative hemoglobin drop, or peri- and postoperative complications was found. The early mobilization was found to not differ significantly between both groups. The data suggest it is safe to perform bilateral simultaneous arthroplasty procedures for hips and knees in the context of a fast-track setting in a specialized high-volume institution. However, further investigations are needed for more precise clinical outcomes in the early postoperative phase, as well as long-term results.

## Figures and Tables

**Table 1:** Characteristics of patients having received a simultaneous arthroplasty.

Pre-Operative Characteristics *n* (%)	THA *n* = 85	TKA *n* = 53	UKA *n* = 22
Age mean (range)	62.8 (32–81)	69.6 (49–84)	65.5 (52–82)
<50	10 (11.76)	1 (1.89)	0 (0)
50–60	27 (31.76)	5 (9.43)	7 (31.82)
61–65	15 (17.65)	9 (16.98)	5 (22.73)
66–70	8 (9.41)	10 (18.87)	3 (13.64)
71–75	11 (12.94)	18 (33.96)	3 (13.64)
76–80	12 (14.12)	6 (11.32)	3 (13.64)
>80	2 (2.35)	4 (7.55)	1 (4.55)
BMI mean (range)	26.3 (18–40)	28.1 (18.9–43.2)	27.7 (22.7–33.5)
<18.5	1 (1.18)	0 (0)	0 (0)
18.5–24.9	35 (41.18)	13 (24.53)	4 (18.18)
25–29.9	35 (41.18)	27 (50.94)	14 (63.64)
30–34.9	8 (9.41)	9 (16.98)	4 (18.18)
35–39.9	5 (5.88)	3 (5.66)	0 (0)
>40	1 (1.18)	1 (1.89)	0 (0)
Female	36 (42.35)	22 (41.51)	3 (13.64)
Male	49 (57.65)	31 (58.49)	19 (86.36)

**Table 2:** Characteristics of patients that received a staged arthroplasty.

Pre-Operative Characteristics *n* (%)	THA *n* = 77	TKA *n* = 64	UKA *n* = 4
Age mean (range)	64 (37–86)	69.7 (51–87)	75.8 (64–81)
<50	8 (10.39)	0 (0)	0 (0)
50–60	21 (27.27)	11 (17.19)	0 (0)
61–65	13 (16.88)	11 (17.19)	1 (25.00)
66–70	14 (18.18)	10 (15.63)	0 (0)
71–75	9 (11.69)	13 (20.31)	0 (0)
76–80	8 (10.39)	14 (21.88)	1 (25.00)
*>*80	4 (5.19)	5 (7.81)	2 (50.00)
BMI mean (range)	26.2 (17.6–39.7)	28.4 (20–42.7)	25.1 (21.4–27.5)
<18.5	1 (1.30)	0 (0)	0 (0)
18.5–24.9	40 (51.95)	21 (32.81)	1 (25.00)
25–29.9	19 (24.68)	18 (28.13)	3 (75.00)
30–34.9	11 (14.29)	18 (28.13)	0 (0)
35–39.9	6 (7.79)	5 (7.81)	0 (0)
>40	0 (0)	2 (3.13)	0 (0)
Female	48 (62.34)	37 (57.81)	0 (0)
Male	29 (37.66)	27 (42.19)	4 (100)

**Table 3:** Demographic data for hip and knee.

Demographic Data	Joint	*p*-Value
Gender	THA	0.017
	TKA	0.116
Age		
<50	THA/TKA	>0.1
50–70	THA/TKA	>0.1
>75	THA/TKA	>0.1

## Data Availability

None.

## Abbreviations

BMI	Body mass index
OCM	Orthopädische Chirurgie München
DVT	Deep venous thrombosis
Hb	Hemoglobin
UKA	unicompartmental knee arthroplasty
LOS	Length of stay
NSAIDs	Nonsteroidal anti-inflammatory drugs
OR time	Operation time
THA	Total hip arthroplasty
TKA	Total knee arthroplasty
